# Supplementary material for: Understanding Biases in Ribosome Profiling Experiments Reveals Signatures of Translation Dynamics in Yeast
Source: PLoS Genet. 2015 Dec 11;11(12):e1005732. doi: 10.1371/journal.pgen.1005732 (PMC4684354; doi:10.1371/journal.pgen.1005732)
Supplement: S1 Text — Details of simulations and analytical model of translation. (PDF) [file pgen.1005732.s001.pdf]

Understanding biases in ribosome profiling experiments reveals signatures of translation dynamics

Jeffrey A. Hussmann*[1], Stephanie Patchett[2], Arlen Johnson[2], Sara Sawyer[2], and William H. Press[1]

[1]Institute for Computational Engineering and Sciences, University of Texas at Austin
[2]Institute for Cellular and Molecular Biology, University of Texas at Austin

November 3, 2015

Supporting information

1 S1 Text

Code

All mapping, simulation, and analysis code is available at `github.com/jeffhussmann`. Software tools used include IPython [23], pysam/samtools [18], numpy [26], scipy [22], matplotlib [11], cython [4], pandas [19], and seaborn [27].

Mapping

All ribosome profiling experiments analyzed involve attaching a known sequence to the 3′ end of RNA footprints to which a reverse transcription primer can be annealed. Some experiments use polyA tailing for this purpose, while others attach an oligonucleotide linker sequence. For experiments using polyA tailing, reads were trimmed from the end back to the first base that wasn't an `A` or an `N`. For experiments using linker sequences, linkers were located in reads by local alignment with the expected sequence and trimmed. Trimmed reads were first mapped to yeast rRNA sequences with bowtie2[16], and any reads that mapped were filtered out. Remaining reads were mapped with tophat2[15] to the yeast genome (version EF4) and spliced transcriptome (using transcript models from the Saccharomyces Genome Database's `.gff` dated `Fri Apr 11 19:50:03 2014`). Unmapped reads had any terminal stretches of `A` trimmed and were put through tophat2 again to recover potential mappings overlapping transcript polyA tails, although this has minimal impact on the analysis presented here. The reverse transcription process used to convert footprints to DNA can add untemplated bases to the end of intermediate antisense DNA products, which ultimately end up located at the beginning of sequencing reads [12]. We observed that the rate at which this happens varies considerably between different experiments. To prevent these untemplated bases from potentially shifting the

*Correspondence: jah@ices.utexas.edu

codon positions that reads end up assigned to, bases that mismatch the reference sequence are trimmed from the beginning of all mappings up to the first matching base. For every annotated coding sequence, uniquely mapped reads of length 28 or 29 were assigned to the in-frame codon closest to the nucleotide at (0-based) offset +15 from the 5′ end of the read; reads of length 30 were assigned to the in-from codon closest to offset +16.

Computing mean relative enrichments

To describe the computation of mean relative enrichments more formally, let g be an index over genes. Let l_g be the length in codons of gene g's coding sequene. Let $c_{g,i}$ be the codon identity at position i in gene g, Let $r_{g,i}$ be the count of uniquely mapped reads assigned to this codon position. Let d be the number of codons to be exclude from the edge of each gene. For each gene consisting of at least $2d+1$ codons, so that there is something left after excluding d from the beginning and the end, compute the mean read count over all eligible positions in a gene

$$M_g = \frac{\sum_{i=d}^{l_g-d} r_{g,i}}{l_g - 2d} \tag{1}$$

and define the relative enrichment at each positions as

$$e_{g,i} = \frac{r_{g,i}}{M_g}. \tag{2}$$

For a given codon identity I and offset F, the stratified set of all eligible positions located exactly that offset downstream of an occurrence of that codon identity is

$$s_{I,F} = \{(g,i) : d < i < l_g - d, c_{g,i-F} = I\}. \tag{3}$$

The mean relative enrichment at the stratified set of such positions is therefore

$$\frac{\sum_{(g,i)\in s_{I,F}} e_{g,i}}{|s_{I,F}|}. \tag{4}$$

When a gene has a small number of reads mapped to it, the denominator in the expression for $e_{g,i}$ is small and the values produced by this expression are noisy. Maximizing signal-to-noise in mean relative enrichments is therefore a balancing act between including as many genes as possible in order to maximize the number of codon positions being averaged over while minimizing the effect of noisy relative enrichment values from lowly-expressed genes. This issue is particularly pronounced in the mean relative enrichment profiles around non-optimal codons (e.g. CGA), for which a disproportionate share of occurrences of the codon identity are in lowly-expressed genes. To navigate this balance, for each experiment, we excluded genes for which $M_g < 0.1$ - that is, genes with an average read density of less than 1 read per 10 codons across the eligible region of the gene. Because the number of useful sequencing reads produced by each experiment varies considerably due to differences in the number of raw reads produced and in the efficiency with which uninteresting rRNA contaminants are removed, the exact set of genes passing this filter varies from experiment to experiment. Profiles of mean relative enrichments in all experiments are qualitatively unchanged but noisier if we instead include every gene with a nonzero number of mapped reads in each experiment.

Simulation details

In order to evaluate the ability of different models of CHX activity to produce patterns observed in experimental data, we developed a simple event-driven simulator of the movement of ribosomes along coding sequences. We made several simplifying assumptions about translation in this simulation. First, we assume the elongation time at each position depends only on the codon identity in the A-site of a ribosome. Second, we assume that the rate of initiation for each mRNA is a constant (but potentially gene-specific) value - that is, we do not model competition for a pool of ribosomes between different mRNAs. Third, we measured time in arbitrary units not grounded in any absolute measurements.

The central object in the simulation is a representation of a single copy of a particular mRNA copy of a coding sequence. For each such mRNA object, multiple ribosomes are tracked as they simultaneous advance along the coding sequence. A priority queue of future events indexed by the time at which each event is scheduled to occur is maintained to determine the ordering of events. Evolution of the system is carried out by popping events off off the priority queue, processing the events, and then inserting any consequent events into the queue.

Simulation for each mRNA object begins with an immediate initiation event at $t = 0$. After each initiation event, the time interval until the next attempted initiation is drawn from an exponential distribution with the rate parameter set to a user specified, potentially gene-specific value. Although we carried out simulations in which the initiation rate of each gene is proportional to the ratio of footprint RPKM to mRNA-seq RPKM from matched experiments (the so-called translational efficiency of the gene [13]), the simulation results shown in the main text have the initiation rate of every gene set uniformly to 0.01. Ribosomes are always assigned to the single codon identity in their A-site, but each ribosome occludes 5 codon positions upstream and 4 codon positions downstream of this. After initiation, the amount of time a ribosome waits at each codon position before attempting to advance is exponentially distributed with a rate parameter determined by the codon identity in the A-site. Ribosomes are prevented from advancing if doing so would cause its A-site to be within 4 codons of the next downstream ribosome's left edge. If this occurs, a new waiting time is drawn, after which the ribosome will attempt to advance again. To efficiently evolve a single instance of a coding sequence to steady state, events are processed until the first ribosome hits the stop codon. If t_{runoff} is the point in time at which this happens, a stopping time is chosen uniformly at random from the interval $[t_{\text{runoff}}, 2t_{\text{runoff}}]$. This stopping time is added to the priority queue as an event, and events are processed until this event is reached.

After steady state is reached, different potential CHX mechanisms can be introduced. Two such mechanisms are considered here. In the first, at the instant CHX is introduced to the system, each ribosome is assigned an amount of time to wait until a CHX molecule first arrives at it and irreversibly halts it. The mean of this waiting time distribution is the mechanistic knob that is assumed to change with CHX concentration. Every ribosome that initiates after CHX is introduced is also assigned a waiting time in the same way. The system is evolved until every ribosome has been arrested and the initiation site is occluded by an arrested ribosome so that no further initiation is possible. The resulting positions of ribosomes are then recorded as simulated read counts. The only way in which this model of CHX action produces sampled positions that differ from the pre-CHX steady state is when stochastic differences in the arrival times of CHX at sequential ribosomes cause

the upstream ribosome to be halted by running into the arrested ribosome in front of it instead of by the arrival of CHX. The average spacing between ribosomes is determined by the ratio between the rate of initiation and elongation rates. The extent to which stalling occurs can be tuned by controlling the ratio between this average spacing and the mean time until CHX arrival. If this ratio is small, pairs of sequential ribosomes frequently experience a large enough difference in CHX arrival times for the trailing ribosome to close the gap between them. This results in spikes in mean enrichment at offsets that are multiples of 10 upstream (i.e. at negative offsets in the profiles of mean enrichment plotted throughout the paper) and broad, low-level enrichment downstream of any slow codon identity but no coherent downstream peaks. Mean enrichments at the A-site experience a contraction towards one, reflecting the fact that the codon identity in the A-site of a ribosome that was stopped by running into the ribosome ahead of it is essentially drawn uniformly from the codon identities in a coding sequence, rather than being drawn in proportion to the elongation times of codon identities. We are unable to find any region of parameter space for which this mechanism produces behavior that qualitatively hints at the changes in active site occupancies and appearance of downstream peaks present in real data.

In the second potential mechanism, at the instant of CHX arrival, the means of the exponential distributions from which the elongation waiting time of ribosomes at each codon identity are changed. Every ribosome with a pending elongation event in the priority queue has this event discarded and redrawn from the new distributions. After this shift in codon-identity-specific elongation rates, a user-specified interval of time is allowed to proceed before the locations of all ribosomes are measured. As discussed in the main text, a potential mechanistic basis for this behavior is that CHX molecules repeatedly bind and unbind from each ribosome, so that the mean time a ribosome spends at a position reflects the influence of the codons located in the ribosome's tRNA binding sites on the rates of CHX association and dissociation.

For either model of CHX action, a template (real) experiment is used to guide the number of simulated reads produced for each gene in order to accurately reflect the dynamic range of expression in the yeast transcriptome. To do this, for each gene, copies of the coding sequence are evolved to steady state and put through simulated CHX treatment before recording simulated read positions until the total number of reads produced for the gene just exceeds the count of reads mapped to that gene in the template experiment.

Modelling transient behavior after changes in relative elongation rates

To analytically model transient patterns in ribosome density following sudden changes in codon-specific relative elongation rates, we assume for simplicity that the amount of time a ribosome spends at a particular position depends only on the identity of the codon positioned at the A-site of the ribosome and that these time intervals are independent and exponentially distributed with a codon-identity specific rate parameter. We also assume that rates of initiation are small enough relative to elongation rates that collisions between ribosomes can be ignored.

Each coding sequence is an ordered sequence of the 61 non-stop codons. Suppose that a particular coding sequence g consists of n codons with identities $\{c_i\}$ for $i = 1, \ldots, n$. Then the life cycle of a ribosome with respect to this coding sequence can be modelled as a

simple continuous-time Markov chain with a dummy state 0 that represents the ribosome doing anything but translating this particular coding sequence. Transition from this state into the act of translating the first codon occurs at some coding-sequence specific initiation rate λ_{init} that is a nuisance parameter for the purposes of this calculation. After this, the ribosome transitions from each codon to the next at a rate determined by the identity of the codon it is currently translating. Assuming that the cell is in a steady state condition, the probability that a random point in time sampled from the lifetime of a ribosome will find it in the act of translating a particular codon, given that it was observed somewhere on this coding sequence, is given by the stationary distribution of this Markov chain, conditional on not being in state 0.

The infinitesimal generator matrix of this Markov chain is

$$\Lambda = \begin{bmatrix} -\lambda_{\text{init}} & \lambda_{\text{init}} & 0 & 0 & \dots & 0 \\ 0 & -\lambda_{c_1} & \lambda_{c_1} & 0 & \dots & 0 \\ 0 & 0 & -\lambda_{c_2} & \lambda_{c_2} & \dots & 0 \\ \vdots & \vdots & \vdots & \vdots & \ddots & \vdots \\ \lambda_{c_n} & 0 & 0 & 0 & \dots & -\lambda_{c_n} \end{bmatrix}. \tag{5}$$

Because this Markov chain is irreducible, it has a unique stationary distribution

$$\mathbf{p}_{\text{steady state}} = \begin{bmatrix} p_0 & p_1 & \dots & p_n \end{bmatrix}. \tag{6}$$

The stationary distribution will satisfy the probability mass-balance equation

$$\mathbf{p}_{\text{steady state}}\Lambda = \mathbf{0}^T \tag{7}$$

and the normalization condition

$$\sum_{j=0}^{n} p_j = 1. \tag{8}$$

It is straightforward to verify that

$$p_0 = \frac{\frac{1}{\lambda_0}}{\sum_{k\in\{0,c_1,\dots,c_n\}} \frac{1}{\lambda_k}} \tag{9}$$

and

$$p_j = \frac{\frac{1}{\lambda_{c_j}}}{\sum_{k\in\{0,c_1,\dots,c_n\}} \frac{1}{\lambda_k}} \tag{10}$$

for $j = 1, \dots, n$ satisfy these equations. To produce the conditional stationary distribution given that not being in the dummy state, simply divide the other components by their sum. The net effect of this is to remove the term corresponding to the dummy state from the denominator, giving

$$p_j = \frac{\frac{1}{\lambda_{c_j}}}{\sum_{k=1}^{n} \frac{1}{\lambda_{c_k}}}. \tag{11}$$

If the probability distribution over states at $t = 0$ is given by $\mathbf{p}(0)$, then it is a standard result that the system of ordinary differential equations governing the flow of probability density between states over time has solution

$$\mathbf{p}(t) = \mathbf{p}(0)e^{t\Lambda}. \tag{12}$$

Consider a coding sequence consisting of 100 copies of codon A, followed by a single copy of codon B, followed by 100 copies of codon A. Suppose that the two codon identities are translated with mean relative elongation times β_A and $\beta_{B,\text{before}}$. Let Λ_{before} be the infinitesimal generator matrix of the Markov chain with these rates, and let $\mathbf{p}_{\text{before}}$ be the steady state distribution under Λ_{before}. Suppose that the system is at steady state and then at time 0 the dynamics of translation are instantaneously changed so that the relative elongation rates of the two codon identities become β_A and $\beta_{B,\text{after}}$. Let Λ_{after} and $\mathbf{p}_{\text{after}}$ be the generator matrix and steady state distribution, respectively, under these new relative elongation rates. Then

$$\mathbf{p}(t) = \mathbf{p}_{\text{before}}e^{t\Lambda_{\text{after}}}. \tag{13}$$

To understand the transient behavior as the system relaxes to the new steady state, decompose $\mathbf{p}_{\text{before}}$ into

$$\mathbf{p}_{\text{before}} = \mathbf{p}_{\text{after}} + (\mathbf{p}_{\text{before}} - \mathbf{p}_{\text{after}}), \tag{14}$$

giving

$$\mathbf{p}(t) = \mathbf{p}_{\text{after}}e^{t\Lambda_{\text{after}}} + (\mathbf{p}_{\text{before}} - \mathbf{p}_{\text{after}})e^{t\Lambda_{\text{after}}}. \tag{15}$$

By construction, $\mathbf{p}_{\text{after}}$ is in the left null space of Λ and is therefore a left eigenvector of $e^{t\Lambda_{\text{after}}}$ with eigenvalue 1 for any t, so this becomes

$$\mathbf{p}(t) - \mathbf{p}_{\text{after}} = (\mathbf{p}_{\text{before}} - \mathbf{p}_{\text{after}})e^{t\Lambda_{\text{after}}}. \tag{16}$$

The left side of this equation represents how much the distribution at time t still differs from the eventual steady state. Except for slight differences in normalization, $\mathbf{p}_{\text{before}} - \mathbf{p}_{\text{after}}$ is essentially an impulse at the location of the single occurrence of codon B, scaled by $\lambda_{B,\text{before}} - \lambda_{B,\text{after}}$. For a particular offset downstream of the occurrence of codon B and a particular value of t, therefore, the linearity of the expression on the right hand side implies that the transient change in magnitude of the downstream wave is proportional to $\lambda_{B,\text{before}} - \lambda_{B,\text{after}}$.

Figures 4 and S8 plot evaluations of this solution at a range of positions around codon B for series of increasing time points for the cases where codon B changes from being slower than codon A to being faster than codon A at $t = 0$ (that is, $\beta_{B,\text{after}} < \beta_A < \beta_{B,\text{before}}$) and where codon B is slightly slower than codon A before $t = 0$ but then becomes even slower (that is, $\beta_A < \beta_{B,\text{before}} < \beta_{B,\text{after}}$), respectively. In a window around codon B of length l on either side of codon B, the instantaneous rate of change in net ribosome density in the entire window is equal to the rate of flow into the leftmost codon position in the window minus the rate of flow out of the rightmost codon position. Until the downstream wave reaches this rightmost position (or any wave of global change in density caused by a relative change in elongation rates compared to the rate of initiation reaches the leftmost position), these two terms remain equal to each other. This implies

that the net density across the window remains unchanged, so the net excess or deficit in the downstream wave must be equal in magnitude but opposite in sign to the change at codon B.

This 'conservation of ribosome density' argument motivates the expectation in Figures 5, S9, and S10 that the downstream wave areas for each codon identity should exactly offset tRNA binding site changes, and the closely related argument that aggregate tRNA binding site enrichments before a CHX-induced change in dynamics can be recovered by adding downstream wave areas back to the binding site enrichments in the presence of CHX. Applying this correction recovers the positive correlations with 1 / tAI expected if codons decoded by less abundant or wobble base-paired tRNAs are on the whole translated slower than average, although a somewhat wide range of positive correlation values are observed across different experiments in Figure 7. While this could represent genuine differences in translation dynamics between the experiments, it seems likely that technical biases could account for much of the variation. When downstream waves have only moved a few codons downstream (as in our experiment), enrichments are affected by biases in how efficiently footprints with different nucleotides at the 5' edge are converted into sequenceable DNA [2]. When waves have moved far enough downstream that large ranges of offsets need to be summed to capture all of their area, patterns in codon usage could lead to small biases in enrichments around different codon identities that aggregate when large ranges of offsets are summed.

References

[1] Frank W. Albert, Dale Muzzey, Jonathan S. Weissman, and Leonid Kruglyak. Genetic Influences on Translation in Yeast. *PLoS Genetics*, 10(10):e1004692, 2014.

[2] Carlo G Artieri and Hunter B Fraser. Accounting for biases in riboprofiling data indicates a major role for proline in stalling translation. *Genome Research*, pages 2011–2021, 2014.

[3] Carlo G Artieri and Hunter B Fraser. Evolution at two levels of gene expression in yeast. *Genome research*, 24(3):411–21, March 2014.

[4] Stefan Behnel, Robert Bradshaw, Craig Citro, Lisandro Dalcin, Dag Sverre Seljebotn, and Kurt Smith. Cython: The Best of Both Worlds. *Computing in Science & Engineering*, 13(2), 2011.

[5] GA Brar, Moran Yassour, Nir Friedman, and Aviv Regev. High-resolution view of the yeast meiotic program revealed by ribosome profiling. *Science*, 335(February), 2012.

[6] Joshua G Dunn, Catherine K Foo, Nicolette G Belletier, Elizabeth R Gavis, and Jonathan S Weissman. Ribosome profiling reveals pervasive and regulated stop codon readthrough in Drosophila melanogaster. *eLife*, 2:e01179, January 2013.

[7] Justin Gardin, Rukhsana Yeasmin, Alisa Yurovsky, Ying Cai, Steve Skiena, and Bruce Futcher. Measurement of average decoding rates of the 61 sense codons in vivo. *eLife*, 3:1–20, January 2014.

[8] Maxim V Gerashchenko and Vadim N Gladyshev. Translation inhibitors cause abnormalities in ribosome profiling experiments. *Nucleic acids research*, 42(17):1–7, July 2014.

[9] Maxim V Gerashchenko, Alexei V Lobanov, and Vadim N Gladyshev. Genome-wide ribosome profiling reveals complex translational regulation in response to oxidative stress. *Proceedings of the National Academy of Sciences of the United States of America*, 109(43):17394–9, October 2012.

[10] Nicholas R. Guydosh and Rachel Green. Dom34 Rescues Ribosomes in 3' Untranslated Regions. *Cell*, 156(5):950–962, February 2014.

[11] John D Hunter. Matplotlib: A 2D Graphics Environment. *Computing in Science & Engineering*, 9(3), 2007.

[12] Nicholas T Ingolia, Gloria a Brar, Silvia Rouskin, Anna M McGeachy, and Jonathan S Weissman. The ribosome profiling strategy for monitoring translation in vivo by deep sequencing of ribosome-protected mRNA fragments. *Nature protocols*, 7(8):1534–50, August 2012.

[13] Nicholas T Ingolia, Sina Ghaemmaghami, John R S Newman, and Jonathan S Weissman. Genome-Wide Analysis in Vivo of Translation with Nucleotide Resolution Using Ribosome Profiling. *Science*, 324(April), 2009.

[14] C. H. Jan, C. C. Williams, and J. S. Weissman. Principles of ER cotranslational translocation revealed by proximity-specific ribosome profiling. *Science*, 2014.

[15] Daehwan Kim, Geo Pertea, Cole Trapnell, Harold Pimentel, Ryan Kelley, and Steven L Salzberg. TopHat2: accurate alignment of transcriptomes in the presence of insertions, deletions and gene fusions. *Genome biology*, 14(4):R36, April 2013.

[16] Ben Langmead and Steven L Salzberg. Fast gapped-read alignment with Bowtie 2. *Nature Methods*, 9(4):357–360, 2012.

[17] Liana F Lareau, Dustin H Hite, Gregory J Hogan, and Patrick O Brown. Distinct stages of the translation elongation cycle revealed by sequencing ribosome-protected mRNA fragments. *eLife*, 3:e01257, January 2014.

[18] Heng Li, Bob Handsaker, Alec Wysoker, Tim Fennell, Jue Ruan, Nils Homer, Gabor Marth, Goncalo Abecasis, and Richard Durbin. The Sequence Alignment/Map format and SAMtools. *Bioinformatics (Oxford, England)*, 25(16):2078–9, August 2009.

[19] Wes McKinney. Data Structures for Statistical Computing in Python. In Stéfan van der Walt and Jarrod Millman, editors, *Proceedings of the 9th Python in Science Conference*, pages 51–56, 2010.

[20] C Joel McManus, Gemma E May, Pieter Spealman, and Alan Shteyman. Ribosome profiling reveals post-transcriptional buffering of divergent gene expression in yeast. *Genome research*, 24(3):422–30, March 2014.

[21] Danny D Nedialkova and Sebastian a Leidel. Optimization of Codon Translation Rates via tRNA Modifications Maintains Proteome Integrity Article Optimization of Codon Translation Rates via tRNA Modifications Maintains Proteome Integrity. *Cell*, pages 1–13, 2015.

[22] Travis E Oliphant. Python for Scientific Computing. *Computing in Science & Engineering*, 9(3), 2007.

[23] Fernando Pérez and Brian E Granger. IPython: A System for Interactive Scientific Computing. *Computing in Science & Engineering*, 9(3), 2007.

[24] Cristina Pop, Silvi Rouskin, Nicholas T Ingolia, Lu Han, Eric M Phizicky, Jonathan S Weissman, and Daphne Koller. Causal signals between codon bias , mRNA structure , and the efficiency of translation and elongation. *Molecular Systems Biology*, pages 1–15, 2014.

[25] Neelam Dabas Sen, Fujun Zhou, Nicholas T Ingolia, and Alan G Hinnebusch. Genome-wide analysis of translational efficiency reveals distinct but overlapping functions of yeast DEAD-box RNA helicases Ded1 and eIF4A. *Genome research*, pages 1–10, 2015.

[26] Stéfan van der Walt, S Chris Colbert, and Gaël Varoquaux. The NumPy Array: A Structure for Efficient Numerical Computation. *Computing in Science & Engineering*, 13(2), 2011.

[27] Michael Waskom, Kyle Meyer, Paul Hobson, Yaroslav Halchenko, Miikka Koskinen, Alistair Miles, Daniel Wehner, Olga Botvinnik, Tobias Megies, Cynddl, Erik Ziegler, Tal Yarkoni, Yury V. Zaytsev, Luis Pedro Coelho, John B. Cole, Tom Augspurger, Diego0020, Travis Hoppe, Skipper Seabold, Phillip Cloud, Stephan Hoyer, Adel Qalieh, and Dan Allan. seaborn: v0.5.0 (November 2014). November 2014.

[28] Christopher C Williams, Calvin H Jan, and Jonathan S Weissman. Targeting and plasticity of mitochondrial proteins revealed by proximity-specific ribosome profiling. *Science*, 346(6210), 2014.

[29] David J Young, Nicholas R Guydosh, Fan Zhang, Alan G Hinnebusch, and Rachel Green. Rli1/ABCE1 Recycles Terminating Ribosomes and Controls Translation Reinitiation in 3'UTRs In Vivo. *Cell*, 162(4):872–84, 2015.

[30] Boris Zinshteyn and Wendy V Gilbert. Loss of a conserved tRNA anticodon modification perturbs cellular signaling. *PLoS genetics*, 9(8):e1003675, August 2013.
