# Supplementary figures and images for: Understanding Biases in Ribosome Profiling Experiments Reveals Signatures of Translation Dynamics in Yeast
Source: PLoS Genet. 2015 Dec 11;11(12):e1005732. doi: 10.1371/journal.pgen.1005732 (PMC4684354; doi:10.1371/journal.pgen.1005732)

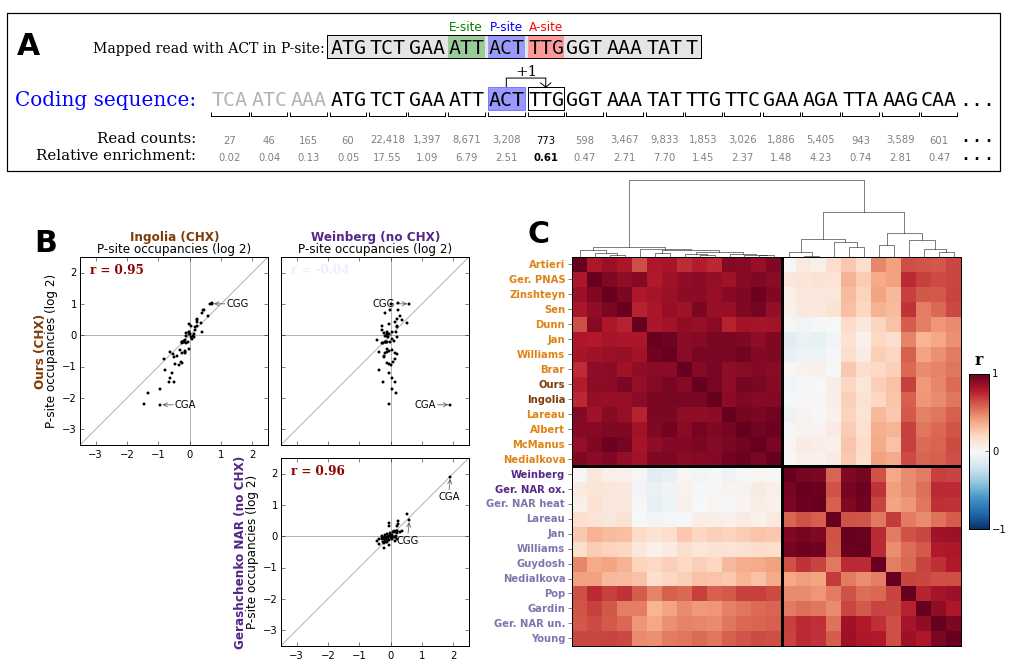

Supplement: S1 Fig — (A) To measure how frequently ribosomes are observed with a particular codon identity (in this example, ACT) in the P-site, the mean of the relative enrichments at all codon positions one codon downstream of an occurrence of the codon identity is computed. Panels (B) and (C) are constructed as in the same panels in Fig 1 but report P-site enrichments. Clustering by P-site occupancy separates CHX (orange) from no-CHX (purple) experiments, but there is substantially less dynamic range in the P-site occupancies of different codon identities in no-CHX experiments than in CHX experiments. Relative P-site occupancies in no-CHX experiments are tightly grouped around one, with the sole exception of CGA, which is consistently a high occupancy outlier. (TIF) [file pgen.1005732.s002.tif]

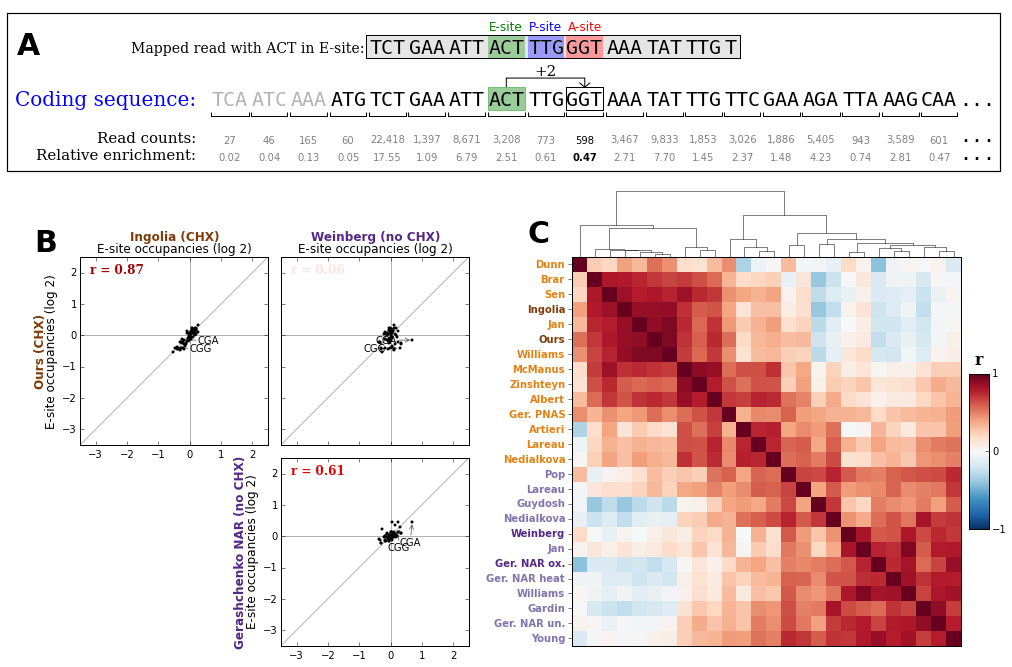

Supplement: S2 Fig — (A) To measure how frequently ribosomes are observed with a particular codon identity in the E-site (in this example, ACT), the mean of the relative enrichments at all codon positions two codons downstream of an occurrence of the codon identity is computed. Panels (B) and (C) are constructed as in Fig 1 but report E-site enrichments. E-site occupancies group weakly by experimental condition but have little dynamic range in either experimental condition and less coherence within experimental condition compared to the A- and P-sites. (TIF) [file pgen.1005732.s003.tif]

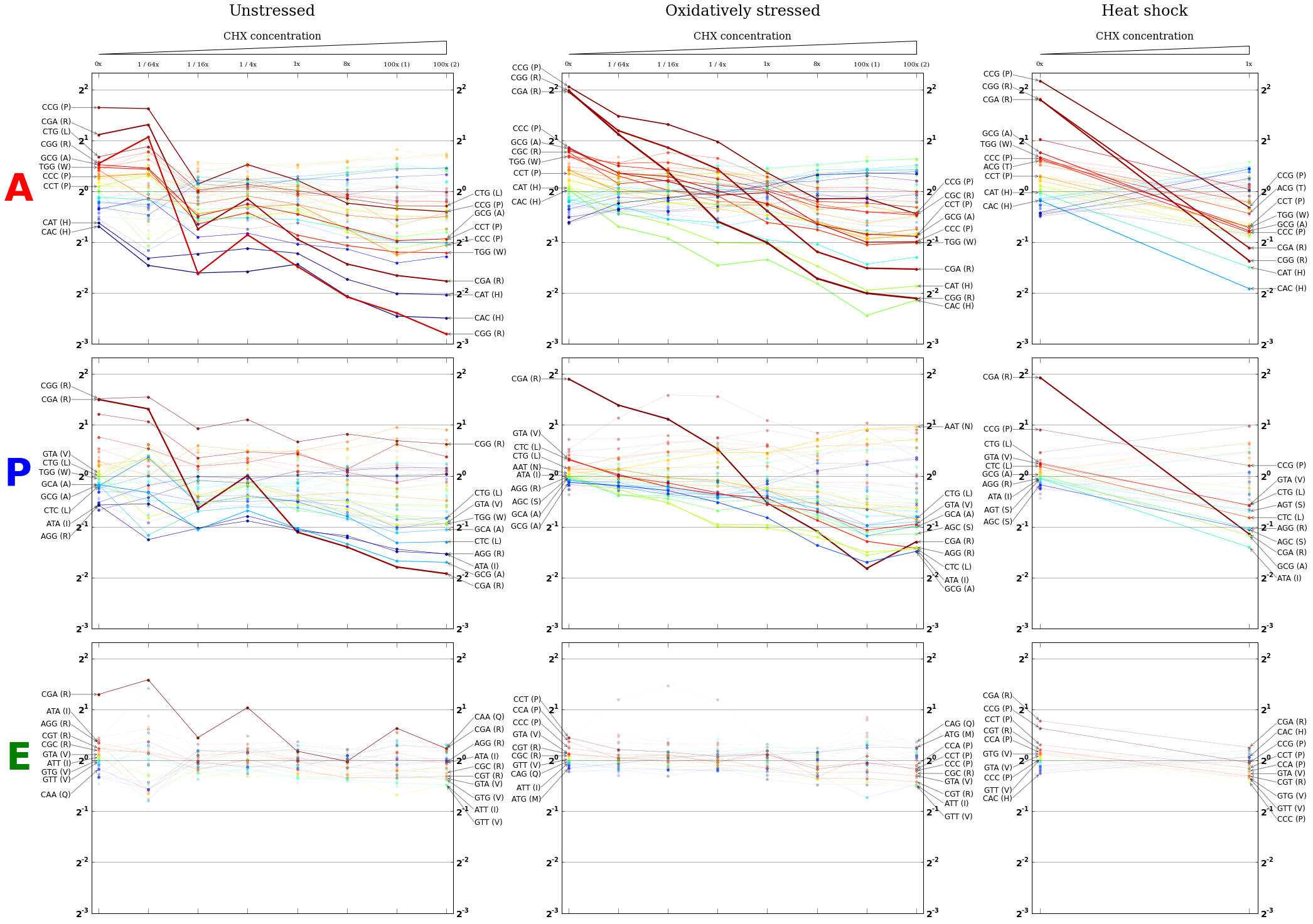

Supplement: S3 Fig — Each panel is constructed as in Fig 2. Each row reports occupancies of a different tRNA binding site (top, A-site; middle, P-site; bottom, E-site). Each column reports occupancies for samples from Gerashchenko [34] under different conditions (left, unstressed; middle, oxidatively stressed; right, heat shock). (TIF) [file pgen.1005732.s004.tif]

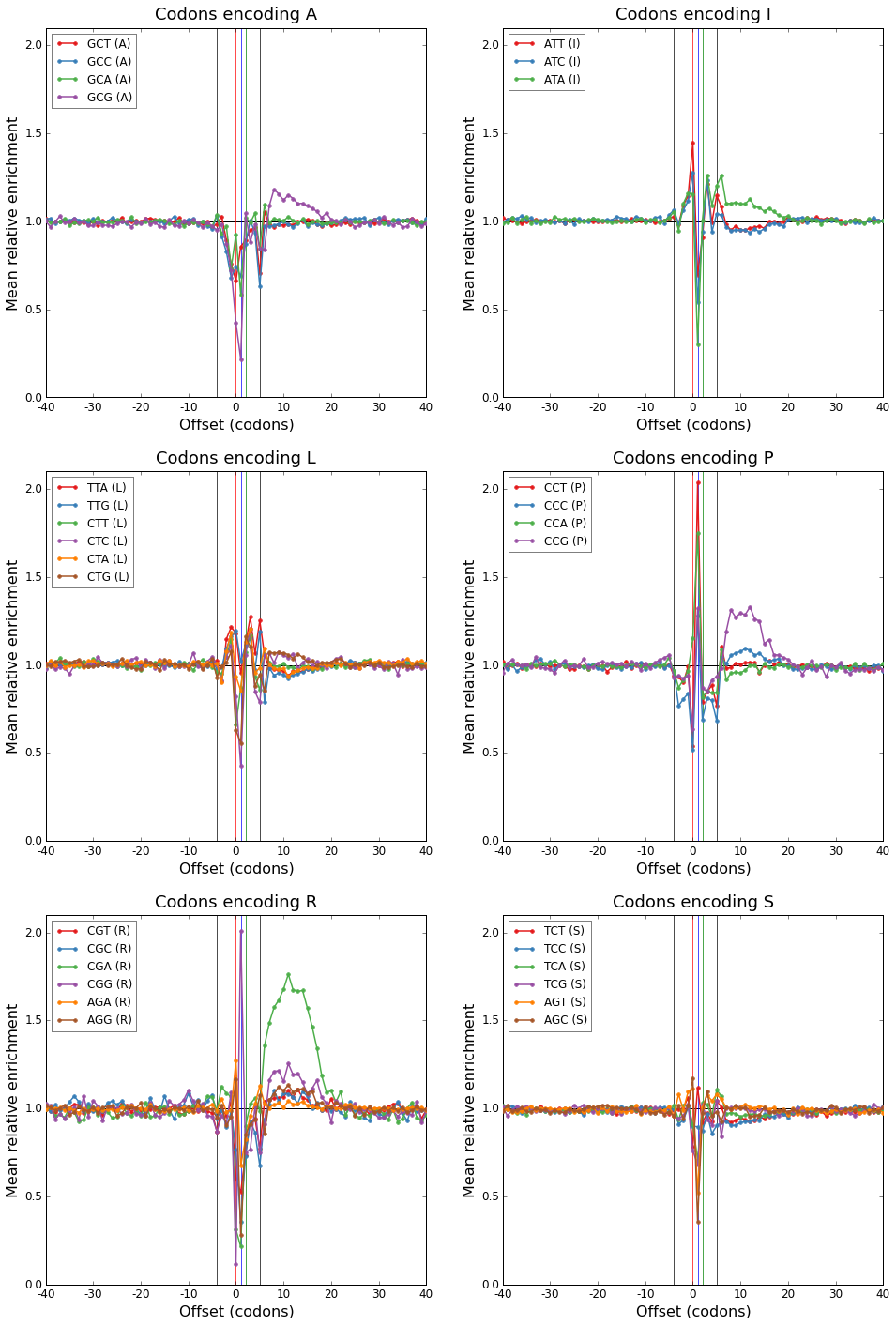

Supplement: S4 Fig — Each panel is constructed as in Fig 3B but shows codons encoding a different amino acid. Several amino acids show substantial variation in the magnitude and direction of downstream peaks between different codons. (TIF) [file pgen.1005732.s005.tif]

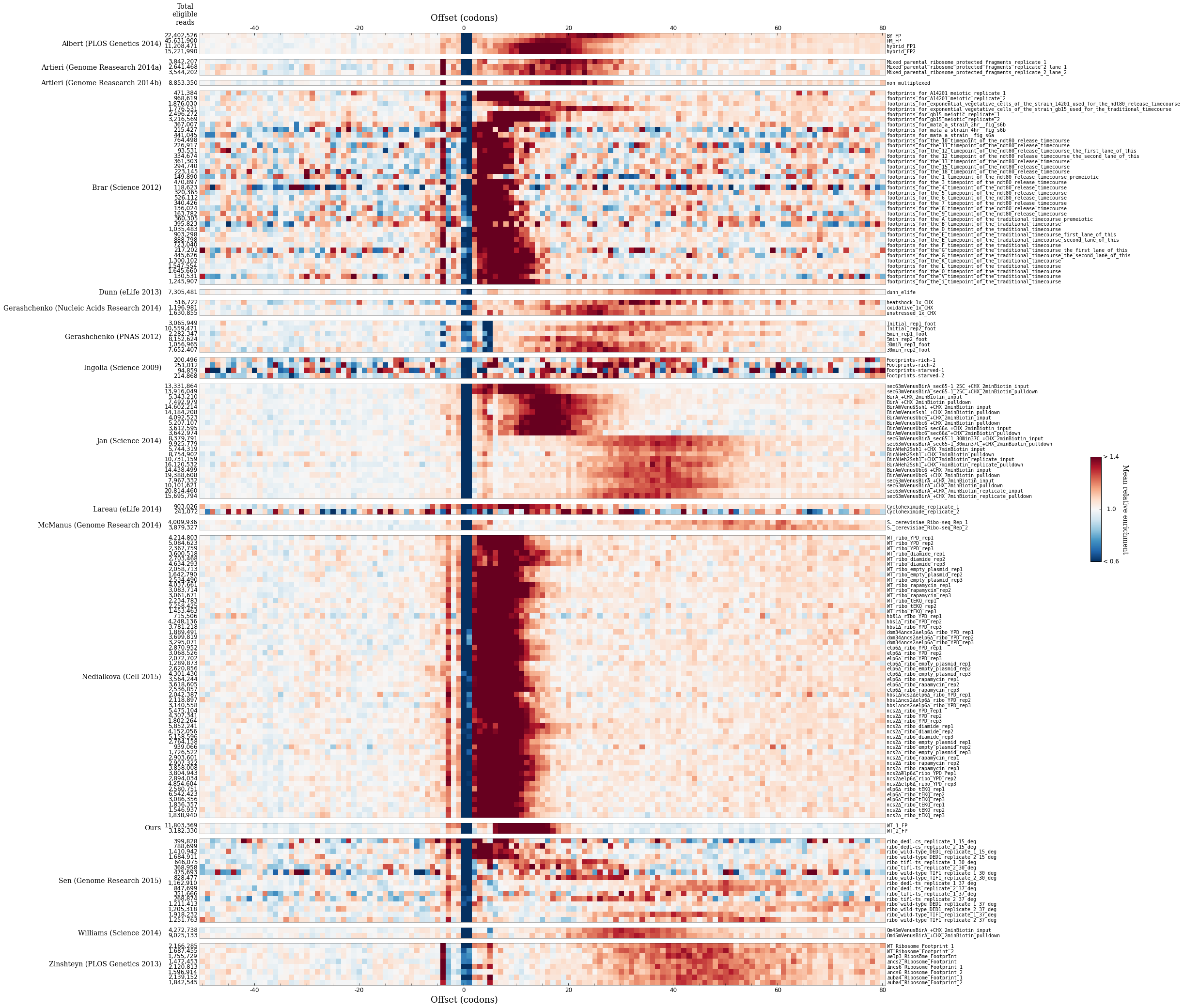

Supplement: S5 Fig — Each row in the heatmap shows mean relative enrichments around CGA in a different experiment, with columns corresponding to different offsets. Experiments are grouped by source study. The number of uniquely mapped reads entering into the computations for each experiment is given on the left. Experiments wtih limited read depth (e.g. those from Ingolia’s original study) exhibit substantially larger offset-to-offset noise in enrichment values. (TIF) [file pgen.1005732.s006.tif]

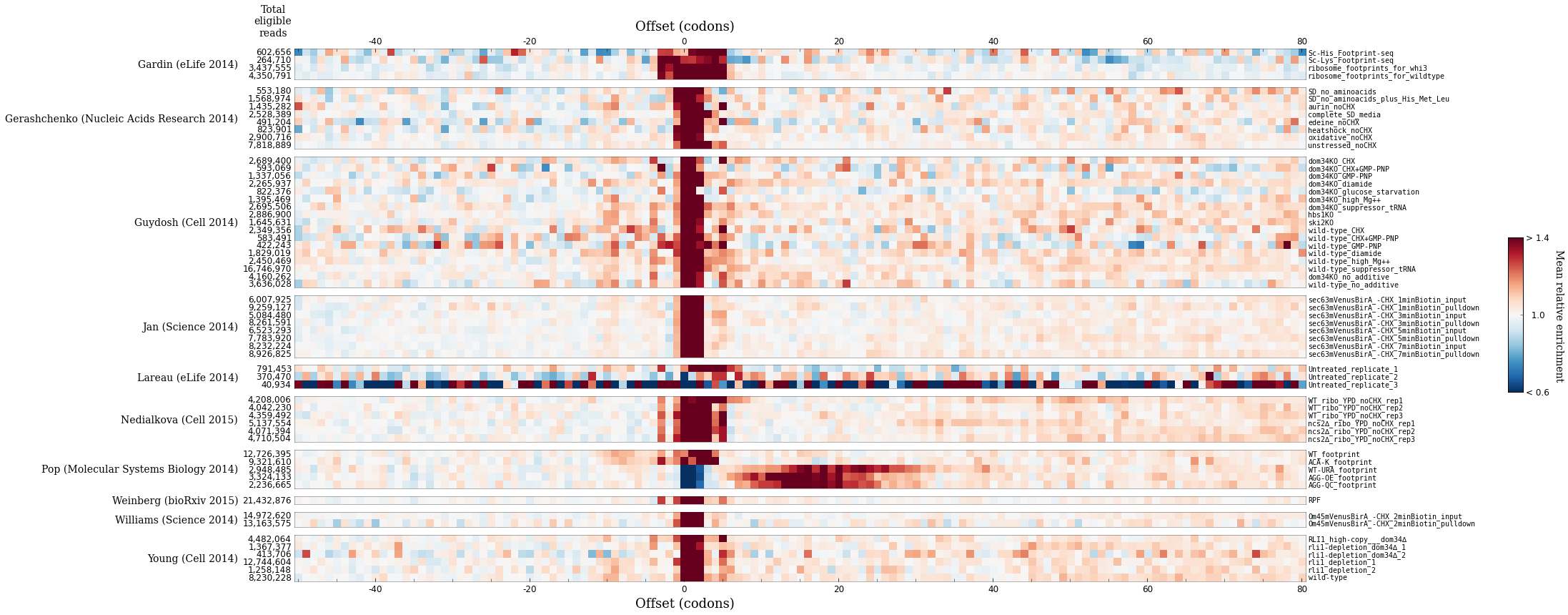

Supplement: S6 Fig — Figure is constructed as in S5 Fig but shows experiments annotated as being performed without CHX pretreatment. Downstream peaks are observed only in three experiments from Pop et al. [36]. (TIF) [file pgen.1005732.s007.tif]

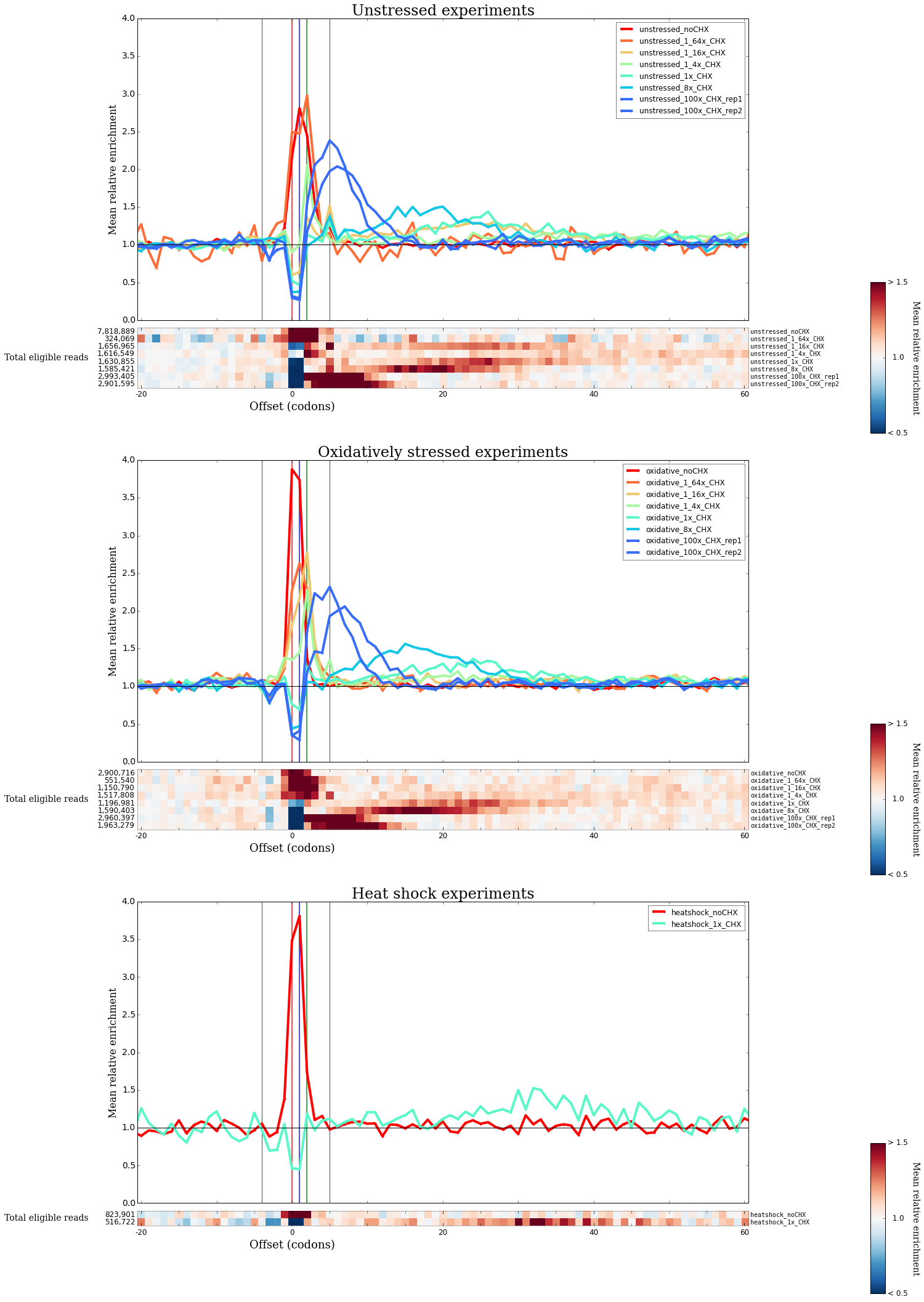

Supplement: S7 Fig — Profiles of mean relative enrichments around CGA for each set of experiments (unstressed, oxidatively stressed, and heat shocked cells) are both plotted and shown in heatmap form. (TIF) [file pgen.1005732.s008.tif]

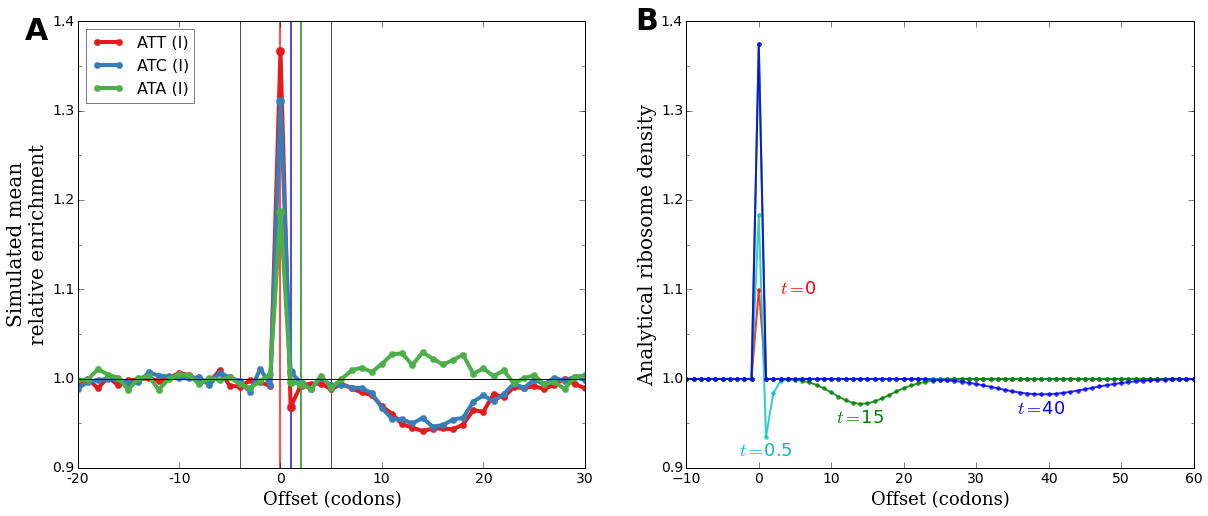

Supplement: S8 Fig — (A) In a simulation of translation, the average relative elongation time of each codon identity was changed from its A-site enrichment in the no-CHX experiment of Weinberg to its A-site enrichment in our CHX experiment. Allowing translation to proceed for a brief period of time under these new dynamics results in a negative peak of depletion downstream of those codons that become relatively slower in the new dynamics, such as ATT and ATC. (B) In an analytical model of the translation of a single special codon surrounded by long stretches of codons that are identically slightly faster than it, changing the dynamics so that the special codon is even slower causes a transient wave of depletion in ribosome density to move downstream from the special codon over time. (TIF) [file pgen.1005732.s009.tif]

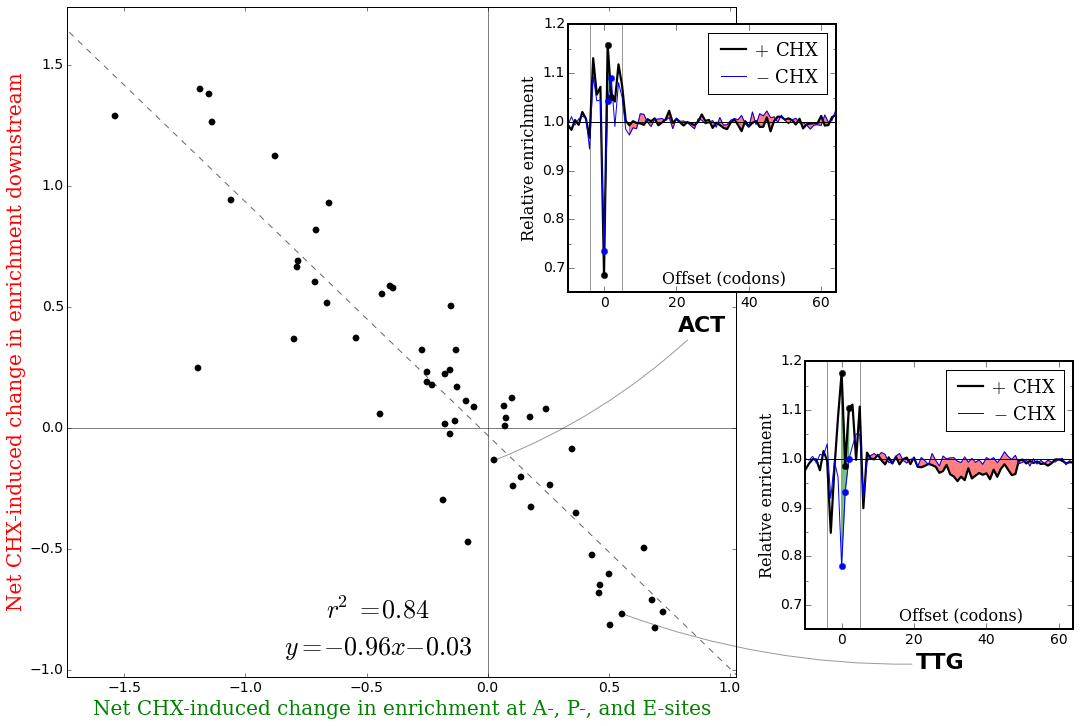

Supplement: S9 Fig — Figure is constructed as in Fig 5 but excludes CGA, CGG, and CCG from the regression. Insets highlight examples of codons with no substantial change (ACT) or a moderate increase (TTG) in net tRNA binding site enrichments in the presence of CHX. The correlation between net tRNA binding site changes and downstream area remains strong (r 2 = 0.84) even after excluding the three codons that participate most in these phenomena. (TIF) [file pgen.1005732.s010.tif]

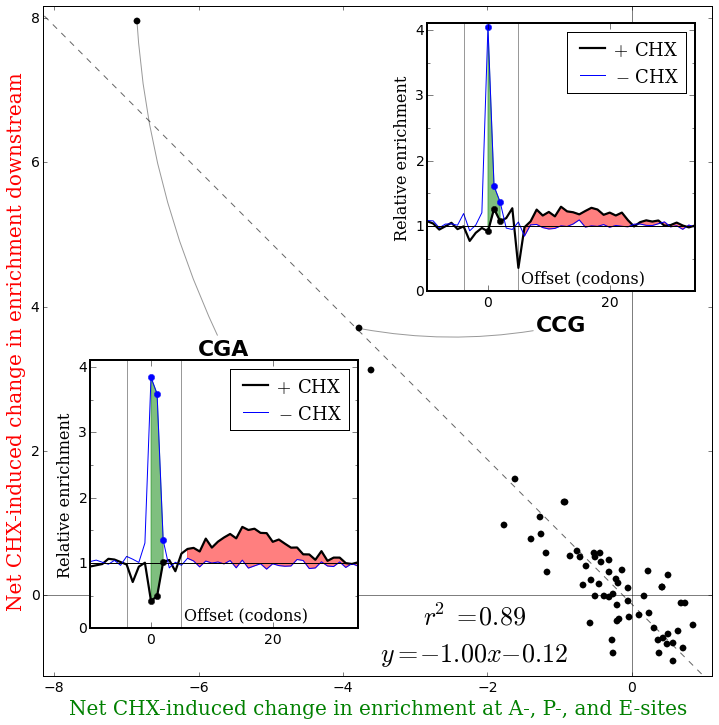

Supplement: S10 Fig — Figure is constructed as in Fig 5 but compares changes between Gerashchenko’s oxidative_noCHX and oxidative_8x_CHX experiments. (TIF) [file pgen.1005732.s011.tif]

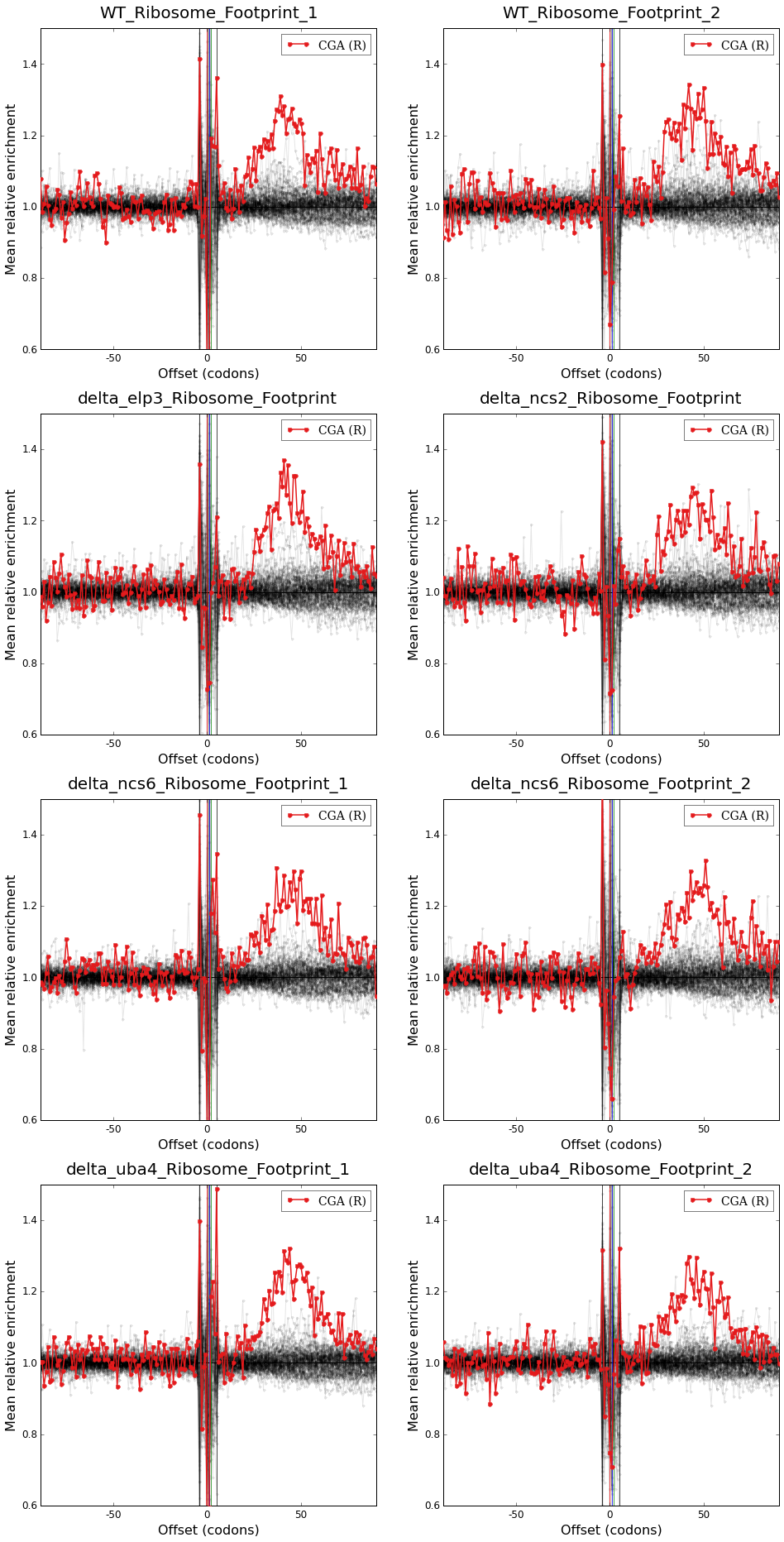

Supplement: S11 Fig — Each panel shows enrichment profiles around all 61 non-stop codons for a particular experiment from Zinshteyn et al. [23], with CGA highlighted in red. All experiments show clear downstream peaks, suggesting that tRNA binding site enrichments no longer reflect in vivo translation dynamics. (TIF) [file pgen.1005732.s012.tif]

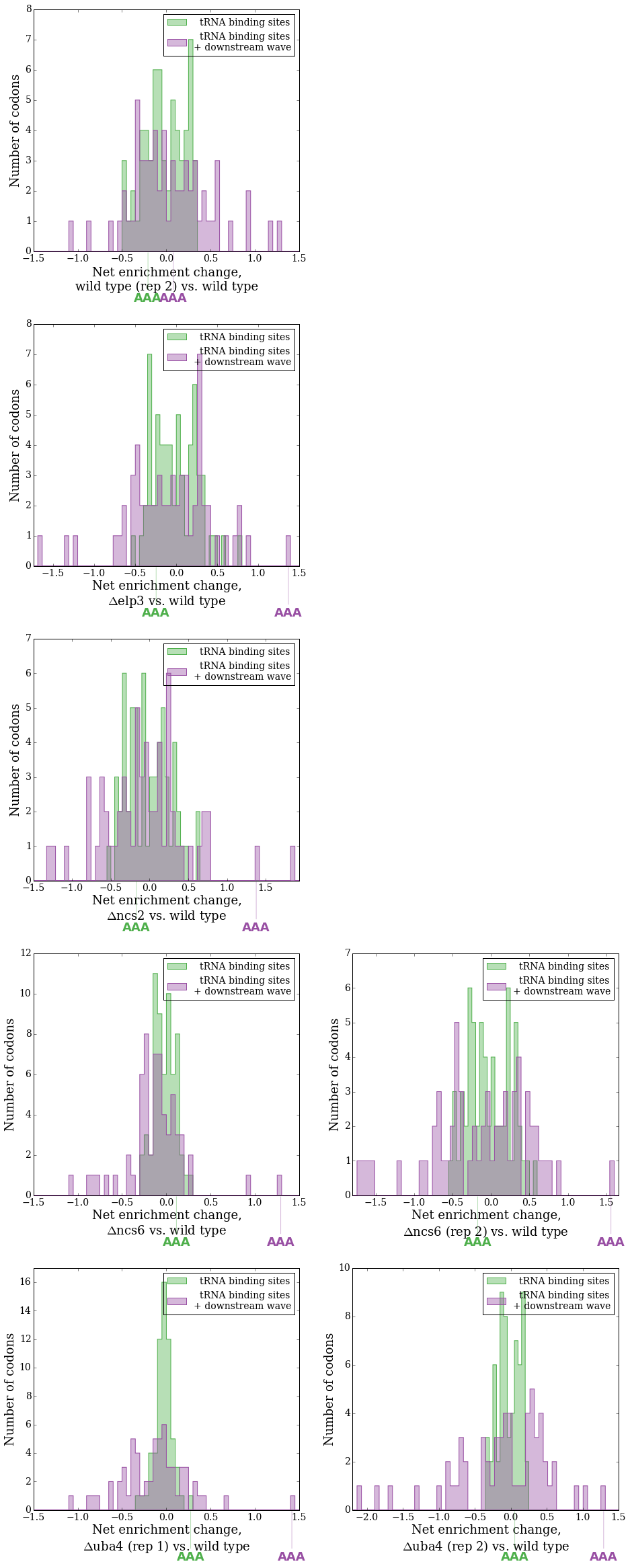

Supplement: S12 Fig — Each panel is constructed as in Fig 8B and shows comparisons between a wild-type experiment and a wild-type replicate (top panel) or different mcm5s2U pathway deletion strains (all other panels). AAA shows a consistently large increase in corrected net enrichment (purple) in every deletion strain but not in the wild type replicate. (TIF) [file pgen.1005732.s013.tif]

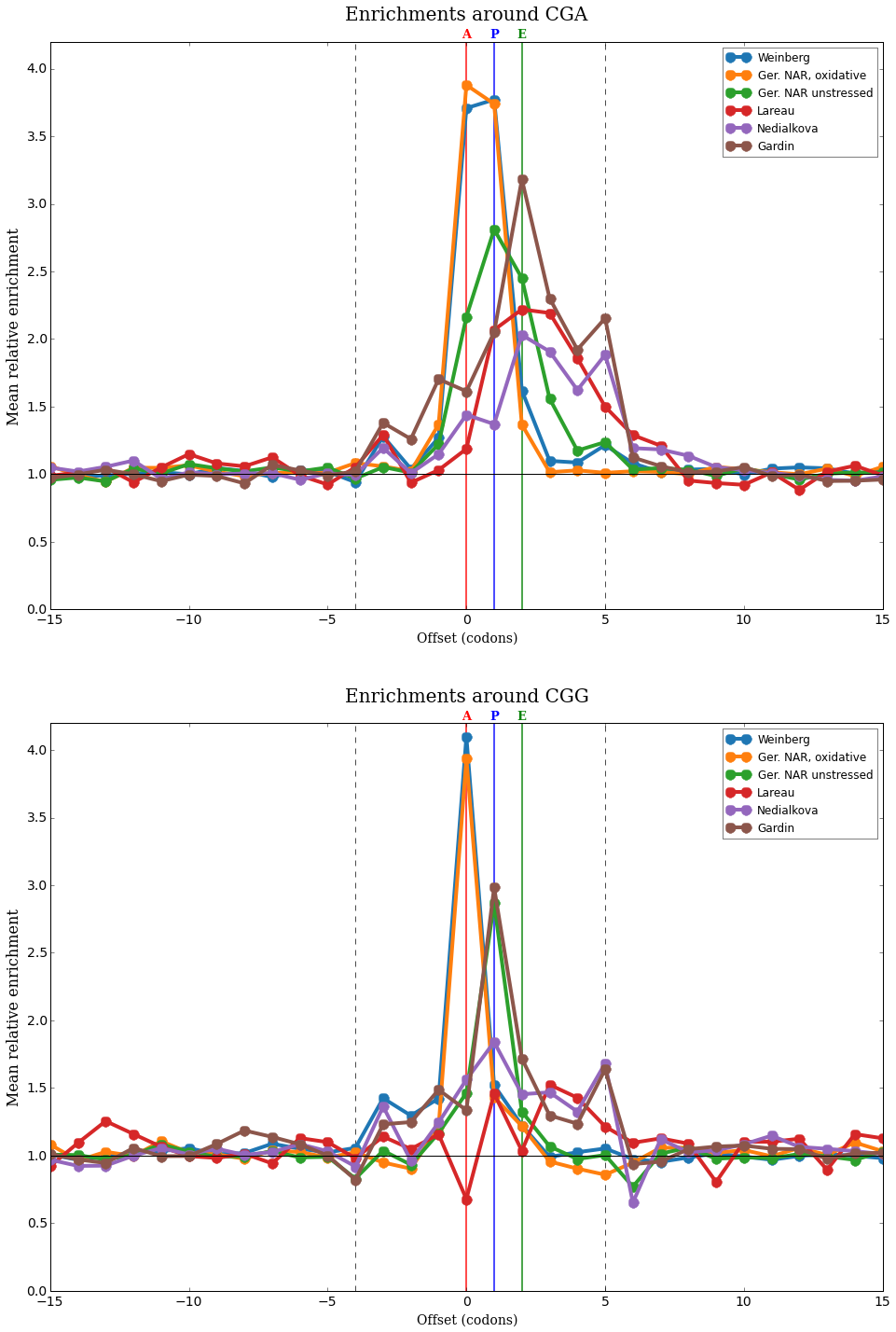

Supplement: S13 Fig — Enrichments around CGA (top panel) and CGG (bottom panel) in the immediate vicinity of the tRNA binding sites in no-CHX-pretreatment experiments from several studies. In data from Weinberg and from Gerashchenko NAR’s oxidatively stressed sample, increased enrichment for CGA is sharply localized to the A- and P-sites, and increased enrichment for CGG is sharply localized to just the A-site. In other experiments, peaks of increased enrichment appear to have moved a few positions downstream. (TIF) [file pgen.1005732.s014.tif]

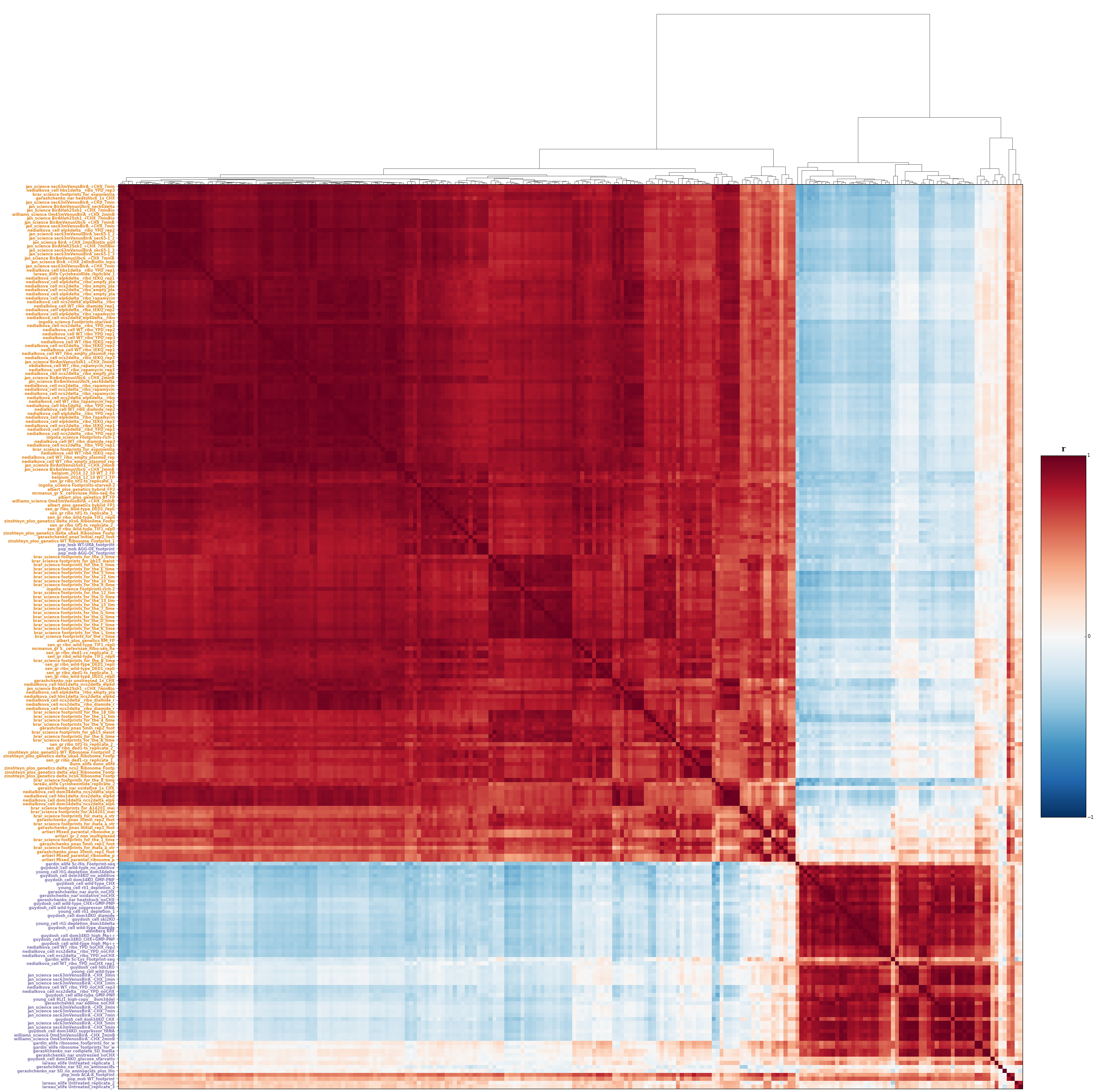

Supplement: S14 Fig — Figure is constructed as in Fig 1C but includes all experiments from each study. Three no-CHX-pretreatment experiments from Pop [36] are more similar to CHX-pretreatment experiments than they are to other no-CHX-pretreatment experiments. (TIF) [file pgen.1005732.s015.tif]

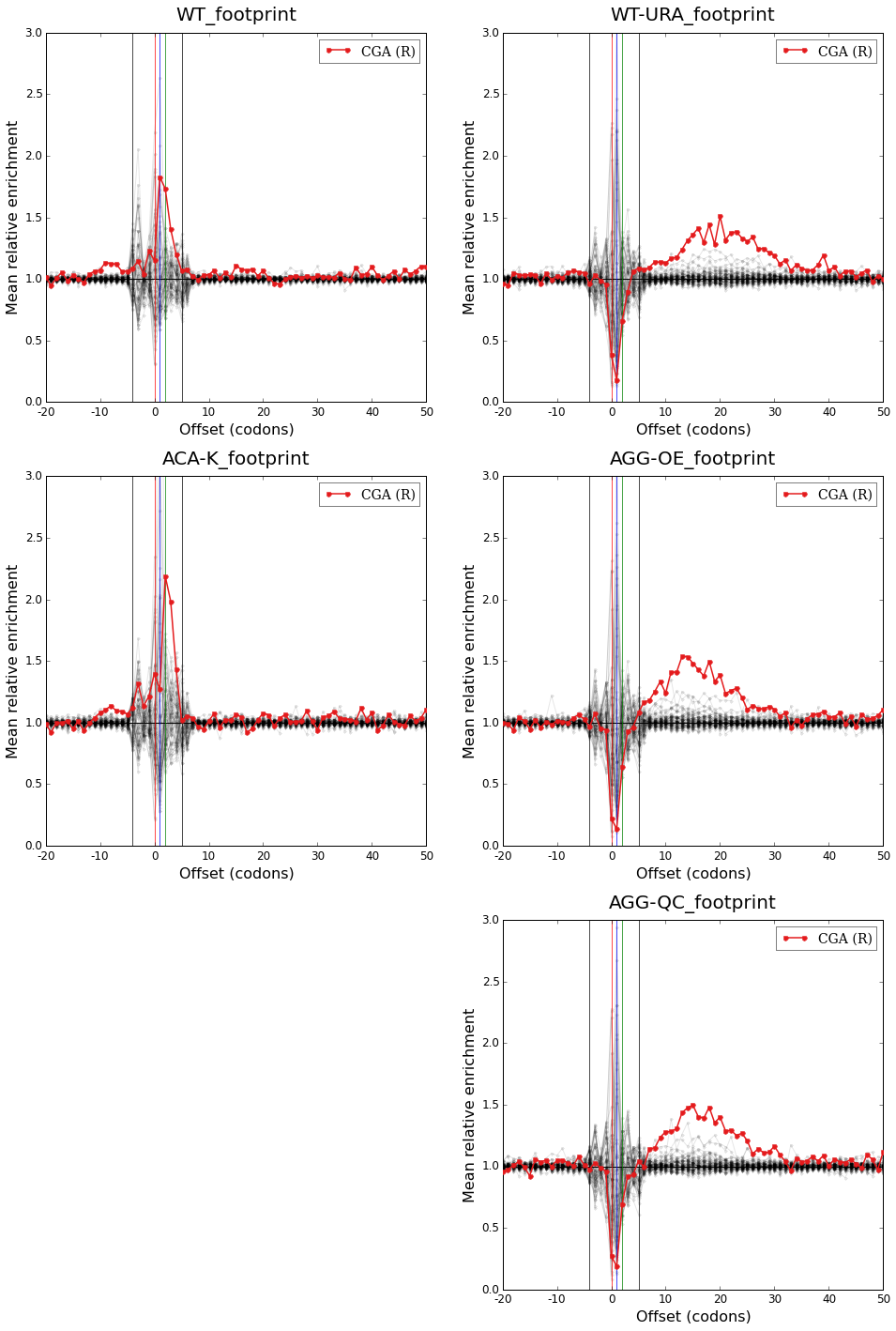

Supplement: S15 Fig — Figure is constructed as in S5 Fig but shows five experiments from Pop [36]. WT-URA_footprint, AGG_OE_footprint, and AGG-QC_footprint all show clear downstream peaks. (TIF) [file pgen.1005732.s016.tif]

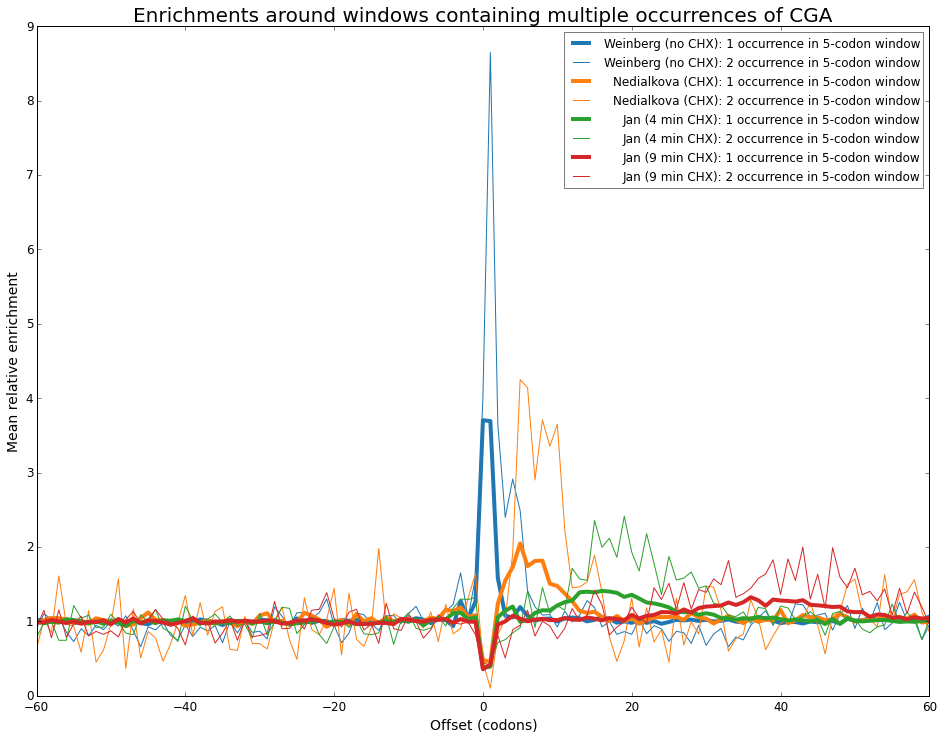

Supplement: S16 Fig — For several different experiments (different colors), mean relative enrichments around all occurrences of CGA that either contain no additional CGAs within the subsequent 4 codons (thick lines) or contain exactly one additional CGA within the subsequent 4 codons (thin lines) are plotted. Peaks downstream of two nearby occurences of CGA are seen to occupy different downstream locations in different experiments and are larger in magnitude than peaks downstream of isolated occurrences of CGA. (TIF) [file pgen.1005732.s017.tif]

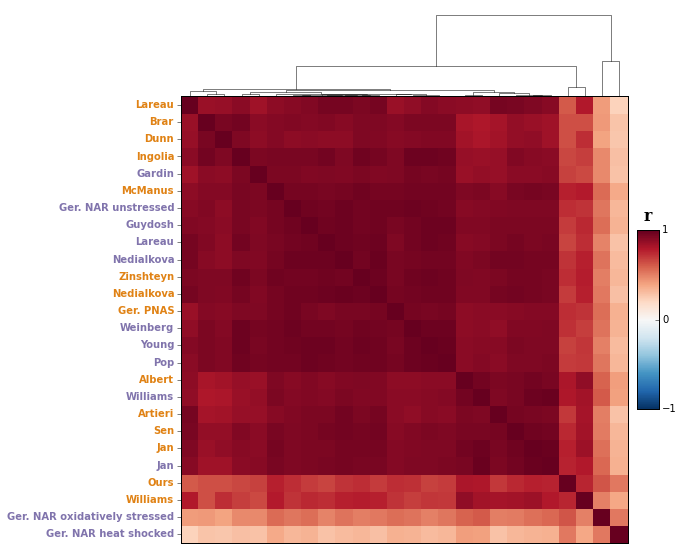

Supplement: S17 Fig — Figure is constructed as in Fig 1C but displays correlations in reads per kilobase per million mapped reads (RPKM) for each gene between experiments. Total ribosome occupancy per gene is not systematically different in CHX-pretreatment experiments (labeled in orange) than in no-CHX-pretreatment experiments (labeled in purple). (TIF) [file pgen.1005732.s018.tif]
