# Supplementary material for: Understanding Biases in Ribosome Profiling Experiments Reveals Signatures of Translation Dynamics in Yeast
Source: PLoS Genet. 2015 Dec 11;11(12):e1005732. doi: 10.1371/journal.pgen.1005732 (PMC4684354; doi:10.1371/journal.pgen.1005732)
Supplement: S1 Table — Accession numbers and sample names for data sets analyzed. (PDF) [file pgen.1005732.s019.pdf]

First author of study	Accession number	Representative sample(s) used
Albert [1]	GSE55400	BY_FP
Artieri [2]	GSE50049	non_multiplexed
Artieri [3]	SRP033885	Mixed_parental_ribosome_protected_fragments_replicate_1
Brar [4]	GSE34082	footprints_for_exponential_vegetative_cells_of_the_strain_gb15_used_for_the_traditional_timecourse
Dunn [5]	obtained from authors	only one sample
Gardin [6]	GSE51164	ribosome_footprints_for_wildtype
Gerashchenko (NAR) [7]	GSE59573	many samples used
Gerashchenko (PNAS) [8]	obtained from authors	Initial_rep2_foot
Guydosh [9]	GSE52968	wild-type_CHX (note: CHX here refers to lysis buffer, not pretreatment)
Ingolia [10]	GSE13750	Footprints-rich-1
Ours	TBD	WT_2_FP
Jan [11]	GSE61012	sec63mVenusBirA_+CHX_7minBiotin_input (CHX)<br>sec63mVenusBirA_-CHX_7minBiotin_input (no CHX)
Lareau [12]	GSE58321	Cycloheximide_replicate_1 (CHX)<br>Untreated_replicate_1 (no CHX)
Mcmanus [13]	GSE52119	S._cerevisiae_Ribo-seq_Rep_1
Nedialkova [14]	GSE67387	WT_ribo_YPD_rep1 (CHX)<br>WT_ribo_YPD_noCHX_rep2 (no CHX)
Pop [15]	GSE63789	WT_footprint
Sen [16]	GSE66411	ribo_wild-type_TIF1_replicate_1_30_deg
Weinberg	GSE53268	Cerevisiae_RPF
Williams [17]	GSE61011	Om45mVenusBirA_+CHX_2minBiotin_input (CHX)<br>Om45mVenusBirA_-CHX_2minBiotin_input (no CHX)
Young [18]	GSE69414	wild-type
Zinshteyn [19]	GSE45366	WT_Ribosome_Footprint_1

Table 1: **Data sources**

References

[1] Frank W. Albert, Dale Muzzey, Jonathan S. Weissman, and Leonid Kruglyak. Genetic Influences on Translation in Yeast. *PLoS Genetics*, 10(10):e1004692, 2014.

[2] Carlo G Artieri and Hunter B Fraser. Accounting for biases in riboprofiling data indicates a major role for proline in stalling translation. *Genome Research*, pages 2011–2021, 2014.

[3] Carlo G Artieri and Hunter B Fraser. Evolution at two levels of gene expression in yeast. *Genome research*, 24(3):411–21, March 2014.

[4] GA Brar, Moran Yassour, Nir Friedman, and Aviv Regev. High-resolution view of the yeast meiotic program revealed by ribosome profiling. *Science*, 335(February), 2012.

[5] Joshua G Dunn, Catherine K Foo, Nicolette G Belletier, Elizabeth R Gavis, and Jonathan S Weissman. Ribosome profiling reveals pervasive and regulated stop codon readthrough in Drosophila melanogaster. *eLife*, 2:e01179, January 2013.

[6] Justin Gardin, Rukhsana Yeasmin, Alisa Yurovsky, Ying Cai, Steve Skiena, and Bruce Futcher. Measurement of average decoding rates of the 61 sense codons in vivo. *eLife*, 3:1–20, January 2014.

[7] Maxim V Gerashchenko and Vadim N Gladyshev. Translation inhibitors cause abnormalities in ribosome profiling experiments. *Nucleic acids research*, 42(17):1–7, July 2014.

[8] Maxim V Gerashchenko, Alexei V Lobanov, and Vadim N Gladyshev. Genome-wide ribosome profiling reveals complex translational regulation in response to oxidative stress. *Proceedings of the National Academy of Sciences of the United States of America*, 109(43):17394–9, October 2012.

[9] Nicholas R. Guydosh and Rachel Green. Dom34 Rescues Ribosomes in 3' Untranslated Regions. *Cell*, 156(5):950–962, February 2014.

[10] Nicholas T Ingolia, Sina Ghaemmaghami, John R S Newman, and Jonathan S Weissman. Genome-Wide Analysis in Vivo of Translation with Nucleotide Resolution Using Ribosome Profiling. *Science*, 324(April), 2009.

[11] C. H. Jan, C. C. Williams, and J. S. Weissman. Principles of ER cotranslational translocation revealed by proximity-specific ribosome profiling. *Science*, 2014.

[12] Liana F Lareau, Dustin H Hite, Gregory J Hogan, and Patrick O Brown. Distinct stages of the translation elongation cycle revealed by sequencing ribosome-protected mRNA fragments. *eLife*, 3:e01257, January 2014.

[13] C Joel McManus, Gemma E May, Pieter Spealman, and Alan Shteyman. Ribosome profiling reveals post-transcriptional buffering of divergent gene expression in yeast. *Genome research*, 24(3):422–30, March 2014.

[14] Danny D Nedialkova and Sebastian a Leidel. Optimization of Codon Translation Rates via tRNA Modifications Maintains Proteome Integrity Article Optimization of Codon Translation Rates via tRNA Modifications Maintains Proteome Integrity. *Cell*, pages 1–13, 2015.

[15] Cristina Pop, Silvi Rouskin, Nicholas T Ingolia, Lu Han, Eric M Phizicky, Jonathan S Weissman, and Daphne Koller. Causal signals between codon bias , mRNA structure , and the efficiency of translation and elongation. *Molecular Systems Biology*, pages 1–15, 2014.

[16] Neelam Dabas Sen, Fujun Zhou, Nicholas T Ingolia, and Alan G Hinnebusch. Genome-wide analysis of translational efficiency reveals distinct but overlapping functions of yeast DEAD-box RNA helicases Ded1 and eIF4A. *Genome research*, pages 1–10, 2015.

[17] Christopher C Williams, Calvin H Jan, and Jonathan S Weissman. Targeting and plasticity of mitochondrial proteins revealed by proximity-specific ribosome profiling. *Science*, 346(6210), 2014.

[18] David J Young, Nicholas R Guydosh, Fan Zhang, Alan G Hinnebusch, and Rachel Green. Rli1/ABCE1 Recycles Terminating Ribosomes and Controls Translation Reinitiation in 3'UTRs In Vivo. *Cell*, 162(4):872–84, 2015.

[19] Boris Zinshteyn and Wendy V Gilbert. Loss of a conserved tRNA anticodon modification perturbs cellular signaling. *PLoS genetics*, 9(8):e1003675, August 2013.
